# Healthcare utilization patterns among migrant populations: Increased readmissions suggest poorer access. A population-wide retrospective cohort study

**DOI:** 10.1016/j.jmh.2025.100340

**Published:** 2025-08-09

**Authors:** Elma Dervić, Ola Ali, Carola Deischinger, Rafael Prieto-Curiel, Rainer Stütz, Ellenor Mittendorfer-Rutz, Peter Klimek

**Affiliations:** a Medical University of Vienna, Center for Medical Data Science, Institute of the Science of Complex Systems, Spitalgasse 23, 1090 Vienna, Austria; b Complexity Science Hub, Metternichgasse 8, 1030 Vienna, Austria; c Supply Chain Intelligence Institute Austria (ASCII), Metternichgasse 8, 1030, Vienna, Austria; d Medical University of Vienna, Department of Internal Medicine III, Clinical Division of Endocrinology and Metabolism, Spitalgasse 23, A-1090 Vienna, Austria; e Department of Clinical Neuroscience, Division of Insurance, Karolinska Institutet, Stockholm, Sweden

**Keywords:** Migration, Healthcare, In-hospital data, Hospitalization Rate

## Abstract

**Background:**

Equal access to health ensures that all citizens, regardless of socio-economic status, can achieve optimal health, leading to a more productive, equitable, and resilient society. Yet, migrant populations were frequently observed to have lower access to health. The reasons for this are not entirely clear and may include language barriers, a lack of knowledge of the healthcare system, and selective migration (a “healthy migrant” effect).

**Objective:**

To examine differences in hospital utilization and readmission rates between Austrian and non-Austrian populations using nationwide hospital claims data, with the aim of disentangling the effects of potential barriers to healthcare access.

**Methods:**

Here, we use extensive medical claims data from Austria (13 million hospital stays of approximately 4 million individuals between 2015 and 2019) to compare the healthcare utilization patterns between Austrians and non-Austrians. We looked at the differences in primary diagnoses and hospital sections of initial hospital admission across different nationalities. We hypothesize that cohorts experiencing the “healthy migrant” effect show lower readmission rates after hospitalization compared to migrant populations that are in poorer health but show lower hospitalization rates due to barriers in access.

**Results:**

We indeed find that all nationalities showed lower hospitalization rates than Austrians, except for Germans, who exhibit a similar healthcare usage to Austrians. Although around 20% of the population has a migration background, non-Austrian citizens account for only 9.4% of the hospital patients and 9.79% of hospital nights. However, results for readmission rates are much more divergent. Nationalities like Hungary, Romania, and Turkey (females) show decreased readmission rates in line with the healthy migrant effect. Patients from Russia, Serbia, and Turkey (males) show increased readmissions, suggesting that their lower hospitalization rates are more likely due to access barriers.

**Conclusion:**

Considering the surge in international migration, our findings shed light on healthcare access, usage behaviours and gender differences across patients with different nationalities, offering new insights and perspectives.

## 1 Introduction

International migrants are nearly 300 million people worldwide, corresponding to approximately 3.6% of the world’s population (United Nations Department of Economic and Social Affairs (DESA), 2020). With an increasing international population living in a different country, certain topics are gaining relevance, particularly their integration into their hosting community (Gnisci, 2008, Niva et al., 2023). Their integration fosters social cohesion and maximizes the contributions of diverse populations to society, enhancing prosperity for all. Successful integration facilitates the smooth adaptation of newcomers to their host country. The integration of migrants involves many social dimensions, including labour, education and health (Gnisci, 2008, Backman et al., 2018, Sîrbu et al., 2020, Ortensi and Ambrosetti, 2022). Depending on the departure and arrival conditions, migrants might easily integrate into their new country or may struggle to access the labour market, could be left out of the education system or might not know how to receive medical care (World Health Organization et al., 2022, Chiarenza et al., 2019, Assi et al., 2019).

Of critical relevance is that migrants tend to have lower access to health services (Brandenberger et al., 2019). For example, data collected in 84 countries between 2018 and 2021 show that in half of the assessed countries, all migrants have access to all government-funded health services under the same conditions as nationals, regardless of their migratory status. In one-third of the countries, access to health services depends on migratory status; in 8% of countries, migrants have access only to emergency health care services; and in 5% of countries, they have no access to any government-funded health services (World Health Organization et al., 2022). Migrants often have unaddressed healthcare needs, particularly in mental and dental health (Lebano et al., 2020, Rousseau and Frounfelker, 2019, Buchcik et al., 2021). It is estimated that migrants in Europe have a high rate of unmet healthcare needs, although there are significant regional disparities (Kullamaa and Reile, 2023, Ward et al., 2019). Even more challenging conditions related to access to healthcare services are experienced by refugee populations. Refugees in Austria rate their health lower than the resident population, predominantly female and Afghan refugees (Kohlenberger et al., 2019). For example, despite having high satisfaction with the Austrian health system, 20% of male and 40% of female refugees report unmet health needs (Kohlenberger et al., 2019). Health inequities of migrants are frequently reinforced by racism, anti-migration bias, healthcare-related prejudice, and unequal treatments (Pattillo et al., 2023).

The inequities in healthcare system access for the migrant population is critical for many reasons. First, it has profound costs in terms of quality of life since some diseases become progressively severe, for example, myocardial infarctions or chronic diseases such as chronic obstructive pulmonary disease (Lewis et al., 2014, Blinderman et al., 2009). Instead of receiving early attention, the burden of the disease, both in terms of health and resources, deteriorates. Second, with less access to healthcare services, patients also have less preventive healthcare, such as early treatment of obesity, vaccinations, etc. Some communicable diseases, such as Influenza, Chickenpox, Hepatitis B and C and sexually transmitted diseases, including syphilis, gonorrhoea, chlamydia, trichomoniasis and HIV, can have a more detrimental impact on communities with less access to the healthcare system and lower health literacy (Quinn and Kumar, 2014).

There are many reasons why migrants often have lower access to health services. For example, even if migrants are legally entitled to certain health services, they often face hidden costs, such as transportation or hiring translators, which reduce their chances of seeking care (World Health Organization et al., 2022). However, the lack of access to health services goes beyond financial costs. For example, in Colombia, health-related expenditures among migrants and non-migrants living with HIV (and in contact with a medical facility) were lower for migrants, with the average per capita expenditure being US$ 859 for migrants compared to US$ 1796 for non-migrants, when adjusted for age and clinical characteristics (World Health Organization et al., 2022). However, the challenge is not only based on offering health services to migrant populations or its cost. Migrants often suffer healthcare discrimination, language barriers and cultural disparities (Gil-Salmerón et al., 2021). Further, the rapid ageing of the world’s population brings new challenges, especially for healthcare systems (World Health Organization, 2015). This demographic shift, characterized by an increasing proportion of elderly individuals, impacts the dynamics of health service demands and resource allocation. Furthermore, longer lifespans are accompanied by a shift in disease burden and a rise in healthcare and long-term care expenses (Eurostat, 2019). An increasing migrant population, combined with an ageing country, requires a fundamental modification of healthcare institutions, practices, and cultural norms to meet these challenges promptly and effectively.

The “‘healthy migrant” effect suggests that migrants might be a particularly resilient population (Ichou and Wallace, 2019, Ma et al., 2020, Holz, 2022, Kennedy et al., 2015). For example, the “healthy migrant” effect is frequently observed with lower mortality rates among migrants (Ichou and Wallace, 2019, Ng, 2011, Razum et al., 1998, Dupre et al., 2012, Holz, 2022). Turkish residents in Germany, for example, have persistently lower overall mortality rates, which extend into the second generation (Razum et al., 1998). However, these studies attribute this to the positive health selection of migrants, meaning healthier individuals are more likely to migrate, potentially beneficial cultural practices, and social support networks. Furthermore, the “salmon bias” hypothesis suggests that the observed lower mortality rates among migrant populations may be partly due to selective return migration. This bias describes older migrants moving back to their birthplace (similar to salmon fish returning to the freshwater streams where they were born to spawn) (Dunlavy et al., 2022, Ma et al., 2020, Holz, 2022).

Observing the links between migration and access to healthcare is quite challenging for many reasons. Firstly, migration is highly dynamic, with repeat and return patterns observed frequently. Some people tend to move and return between different locations frequently, thus spending only a limited amount of time in any location (Prieto-Curiel et al., 2022). Recognizing these variations in migration patterns is crucial for understanding how migrants interact with the healthcare system. For instance, individuals who spend extended periods in a host country are more likely to establish regular healthcare-seeking behaviours and utilize a broader range of healthcare services compared to those with shorter stays. Therefore, accurately assessing healthcare utilization among migrants requires accounting for the diverse staying time in a host country and considering individual differences based on factors like gender, age, and country of origin. Secondly, there are many differences between the migrant and the residence population, including age structure, fertility rates and others. Finally, both migration and healthcare information are very sensitive data and subject to strict privacy regulations that are difficult to manipulate at large scales.

The inequities in healthcare access are particularly critical for Austria. One in five persons living in Austria is a migrant (residents in Austria without Austrian citizenship), with an even higher ratio in larger cities like Vienna (Prieto-Curiel et al., 2024). In this study, migrants are defined by foreign nationality, a criterion unaffected by naturalization rates (which in Austria remained below 1% from 2015 to 2019) (Statistik Austria, 2024a). We explored how nationality influences hospitalization and readmission rates among patients within the Austrian healthcare system. We hypothesized that significant disparities and patterns in inpatient healthcare access and utilization vary across different national backgrounds and that some nationalities have limited access to the healthcare system, worsening their quality of life. In particular, we hypothesize that readmission rates might uncover one of two reasons for decreased healthcare utilization: the “healthy migrant” effect and cultural barriers. Cohorts with the healthy migrant effect will exhibit lower admission and readmission rates post-hospitalization. In contrast, migrant populations with poorer health may have lower hospitalization rates due to access barriers, leading to higher readmission rates after initial hospitalization.

We analysed data from an electronic health registry covering almost four million residents of Austria, documenting over 12 million in-hospital stays spanning five years from 2015 to 2019 to test these hypotheses. A unique aspect of this dataset is the inclusion of patients’ nationality information, enabling us to differentiate and compare the inpatient healthcare utilization patterns between Austrians and non-Austrians. Furthermore, this allowed us to explore the healthcare system usage patterns of migrant patients in Austria, offering valuable insights into the healthcare dynamics within diverse population segments. We introduced the hospitalization rate to quantify how often different people use inpatient care, and the readmission rate, defined as the probability of patients returning to the hospital within a given time window following their initial visit (Milne and Clarke, 1990). Hospital readmission rates can be used as an indicator of the quality of care (Pugh et al., 2021, Kristensen et al., 2015). For example, using the Nationwide Readmissions Database, it was found that increased social needs correlate with higher readmission rates (Bensken et al., 2021). Here, we account for individual differences in the duration of migrants’ stays by introducing their Years of Life (YoL) in Austria, where YoL=1 one for an entire year spent in the country and decreases proportionally with less time spent. Even though, in Austria, over 99% of legal residents have health insurance coverage (Vintila and Lafleur, 2020), we observed that migrants, particularly from remote countries, are less likely to gain access to health services.

## 2 Methods

### 2.1 In-hospital data

The analysed dataset captures a comprehensive overview of hospital admissions in Austria, encompassing around 13 million cases from four million people (N=3,999,832) from January 2015 to December 2019. The demographic composition of this population is balanced in terms of gender (with 46% male and 54% female patients). It has records of each hospitalization, including unique patient identifiers, sex and age (in a resolution of five years), along with primary and secondary diagnoses, the dates of admission and discharge, the nature of the discharge (such as standard release or transfer to another facility, etc.), region of residence, region of hospital (Haug et al., 2020, Dervic et al., 2021), and nationality of the patients. While the primary diagnosis represents the main reason for hospitalization, any additional diagnoses during the stay are recorded as secondary.

Primary and secondary diagnoses in this dataset are classified using level-4 ICD10 codes, the global standard for medical coding and ensuring consistency across healthcare systems (World Health Organization, 2019). The International Classification of Diseases (ICD) is a system produced by the World Health Organization that is crucial for tracking health trends and compiling international health statistics. This dataset has 12040 distinct medical diagnoses, all classified under ICD codes. The five most frequently occurring primary diagnoses in ICD10 coding within this dataset are detailed in SI Figure 5.

**Fig. 1:**
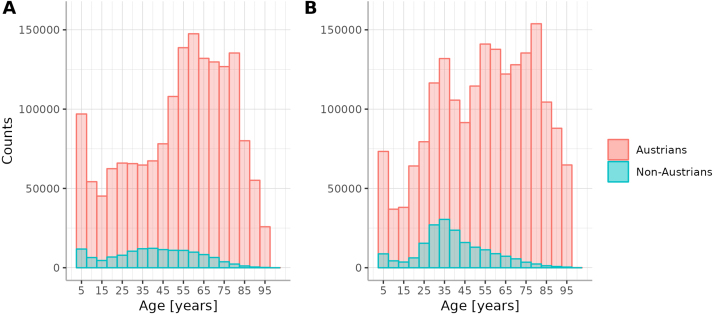
Age distribution (a) males (b) females.

The mean age of the whole cohort is 52.8 SD 24.4 years. Yet, there are differences across nationalities. For Austrian patients, the mean age is 53.9 SD 24.4 years, and for non-Austrians, it is 40 SD 18 years, Fig. 1.

**Fig. 2:**
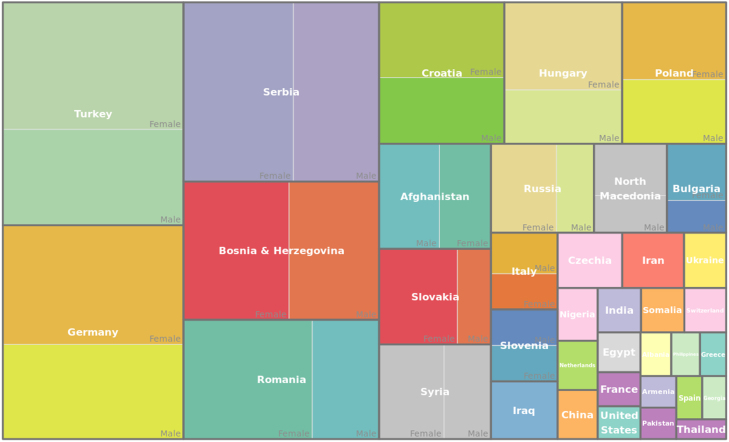
Proportion of each country of origin in migrant patients.

We excluded all patients with more than one nationality from the analysis, which was 1.63% of all patients. In the analysed cohort, patients whose nationality is not Austrian comprise 379,854 individuals, Fig. 2, accounting for 9.4% of the entire cohort. Within this subset, females represent 57.7%, while males constitute 42.3%. The majority of these countries have a higher proportion of female patients compared to male patients. Specifically, there are 32% more female patients of Turkish origin than their male peers, 26% more from Germany, and 28% more from Serbia. Notably, among the top ten countries of origin with the largest patient numbers, the cohort from Afghanistan is an exception, exhibiting 15% fewer female patients compared to males of the same nationality. The female cohort from Afghanistan makes up around 35.8% of the Afghan population, with around 19795 females compared to 35365 males (see Table 1).

**Fig. 3:**
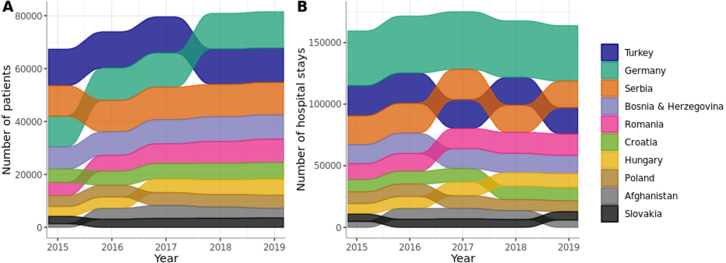
Number of (a) patients and (b) hospital stays across top ten counties of origin, determined by the patient counts from in-hospital data.

Non-Austrian patients had approximately 1.3 million stays, which is 9.79% of all recorded stays, from which 46.31% stays of males and 53.69% of females, Fig. 2. While Germany ranks second in terms of patient count, it significantly leads as the country with the highest number of hospital stays, Fig. 3.

**Table 1:** Total number of patients, percentage of the non-Austrian cohort, Ratio of number females and males for different countries of origin. Extended table with all countries available in Supplementary Information, Table 1.

Country	ISO2	Total	Percentage	Ratio of females and males
Turkey	TR	45 463	11.43	1.32
Germany	DE	43 610	10.97	1.26
Serbia	RS	39 580	9.95	1.28
Bosnia & Herzegovina	BA	30 514	7.67	1.17
Romania	RO	26 355	6.63	1.93
Croatia	HR	20 006	5.03	1.14
Hungary	HU	18 816	4.73	1.63
Poland	PL	16 613	4.18	1.20
Afghanistan	AF	13 270	3.34	0.86
Slovakia	SK	12 076	3.04	2.34

Austria	AT	3 669 104		1.15

### 2.2 Estimating the years of life spent in the country

We take into account that some migrants spend most of their time in the host country, but others arrive late (for example, students who frequently arrive during the summer break), and others tend to spend only part of their time in the host country. To account for these individual differences, we calculate the years of life spent by a person YoL such that YoL=1 if a person spends the entire year in Austria and has a smaller value with less time spent in the country. This measure is similar to the person-years measure used in migration studies, which accounts for the time spent by migrants when analysing outcomes such as economic earnings and mortality (Constant and Massey, 2003, Wallace and Kulu, 2015). The YoL of a non-Austrian resident depends on the country of origin, age and sex, so we model the YoL estimates as a function of those variables. We assume the YoL pattern is conserved per age group, sex, and country between 2023 and 2019 (details in the SI). We calculate the YoL spent by non-Austrian residents by using the YoL ratio across the country, sex, and age of 2023, and we apply it to the non-resident population in 2019 (details in the SI).

**Fig. 4:**
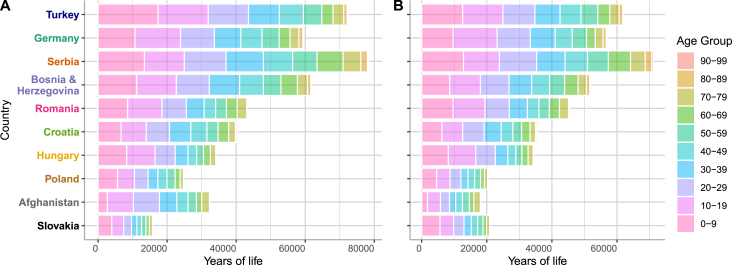
Estimated number of years of life from the top ten countries, determined by the patient counts from in-hospital data in 2019 in Austria (a) males (b) females. Years of life are equivalent to the number of people from each country of origin if they spend all of the year in Austria, and a fractional number of the people if they spend less than a year (details in the SI).

Data on the number of days spent by residents in Austria per age, sex, and country of origin was provided by the Federal Ministry of Interior of Austria, Bundesministerium für Inneres (BMI) for 2023 (Prieto-Curiel et al., 2024). Among the 1.6 million residents with non-Austrian nationality, 49% are female and 51% are male. The average age among these residents is 39.75 years, with females averaging 40.16 years and males 39.37 years, Fig. 4. Data on the whole population of Austria and residents with non-Austrian nationality for 2015 to 2019 is provided by Statistik Austria via the online WEB application STATcube - Statistische Datenbank (Statistik Austria, 2024b).

### 2.3 First hospital admissions

**Fig. 5:**
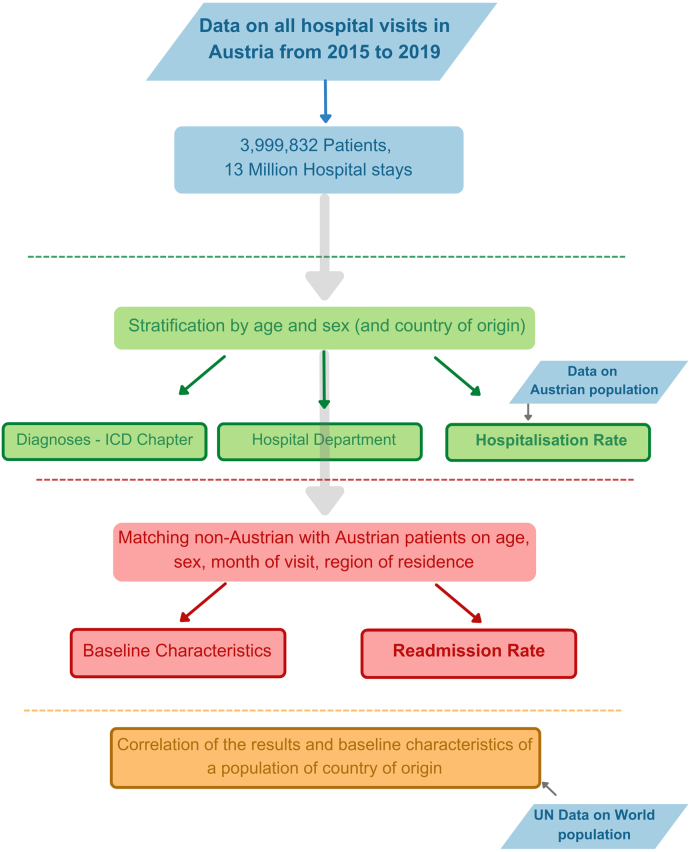
Workflow of the research presented in this article.

We analysed in-hospital data, concentrating specifically on patients’ first hospital admissions, Fig. 5.

We examined the primary diagnoses as the reason for hospital visits. Primary diagnoses in this dataset are coded using the level-4 ICD10 codes. We organized all ICD10 4-digit diagnosis codes into ICD Chapters (based on the first letter of each code), resulting in a total of 21 chapters. All ICD10 chapters are listed for better visibility in Supplementary Information Table 2. We then calculated the prevalence of each primary diagnosis chapter across each 10-year age group, sex, and nationality. Furthermore, to assess the differences in these probabilities across various cohorts, we employed the two-proportions z-test. This test’s null hypothesis states that the respective probabilities for Austrian and non-Austrian patients are identical. The null hypothesis is rejected for cases where p_value<0.05. Subsequently, we calculated the ratio of these prevalences to the ones observed in Austrian patients for cases where we found a significant difference (p_value<0.05). This allowed us to draw comparisons between non-Austrian and Austrian patients in terms of the reason for their initial hospital admissions.

Moreover, the data shows 38 different hospital departments, such as Internal Medicine, Trauma Surgery, Dermatology, and Venereology (All sections are listed in SI, Table 3). We extended our analysis to include the prevalence of admissions in each hospital session (proportion of individuals admitted in each hospital session within an analysed timeframe), stratifying the data by 10-year age groups, sex, and nationality.

We introduced the hospitalization rate to quantify how often cohorts of patients with different nationalities utilize (at least once) inpatient care. This rate is determined by dividing the number of hospitalized patients by years of life (approximation for the number of residents) in each specific age group, sex, and nationality category during 2019. We calculated the average hospitalization rate for each nationality by computing a weighted average across age groups. The weights were assigned proportionally to the frequency of the general population within each age group. Additionally, for any group (stratum) with fewer than 50 patients, we replaced its hospitalization rate with the corresponding rate for Austrian patients.

To accurately compare non-Austrian cohorts with their Austrian counterparts, we matched groups based on age, sex, the month of the hospital visit, and patient region in a ratio of 1:1. Matching controls for key confounders, ensuring that comparisons between groups reflect true differences rather than due to these potential confounding factors. Following this matching process, we calculated the average number of hospital days, the number of hospital stays, and the number of diagnoses for each matched cohort during the observed period. Additionally, we tested if these estimates are significantly different for specific nationalities and its matched cohorts of Austrian patients, using independent samples T-Test (with a p_value<0.05).

The readmission rate is defined as the probability of patients returning to the hospital within a given time window following their initial visit. It is often used as a quality indicator in healthcare, with a high readmission rate potentially signalling issues in patient care. It can also reflect broader systemic problems, like lack of access to health care, which can slow a patient’s recovery. Hence, a high readmission rate can indicate patients coming to the hospital too late with severe conditions. For our analysis, we calculated this rate for patients from different countries and their matched Austrian cohorts and for time windows ranging from three months to one year. To draw comparisons, we then computed the ratio of each country’s readmission rate to the readmission rate of its corresponding matched Austrian cohort.

## 3 Results

### 3.1 Matching population groups

We found substantial differences in hospitalization rates (reflecting inpatient care utilization) and readmission rates (measuring how often patients are readmitted to the hospital within the next year) across cohorts of patients with different nationalities. In Austria, where nearly 20% of the population are migrants, patients whose nationality is not Austrian comprise less than 10% of all hospital patients and less than 10% of the number of hospital nights. To consider varying age and sex- and gender-related structures, we matched different population cohorts (see the Methods section). Female patients of Austrian nationality have a hospitalization rate of 0.22, and males have a rate of 0.23.

Female Syrians have a 1.5-fold (0.33) increased hospitalization rate, whereas, for Russian females, the rate decreases by 50% (0.12). However, hospital rates not related to pregnancy are significantly different for some nationalities. For instance, for female Syrians, it is 0.21. The highest hospitalization rate among males is for patients from Austria, and the lowest hospitalization rate among males is for patients from North Macedonia, which is 50% smaller compared to Austrians, with a hospitalization rate of 0.12. Looking at the top ten countries of origin with the most extensive diaspora in Austria, we could see that for German patients, females have a rate of 0.25, and males have a rate of 0.21, so they have a similar hospitalization rate to their Austrian counterparts.

**Fig. 6:**
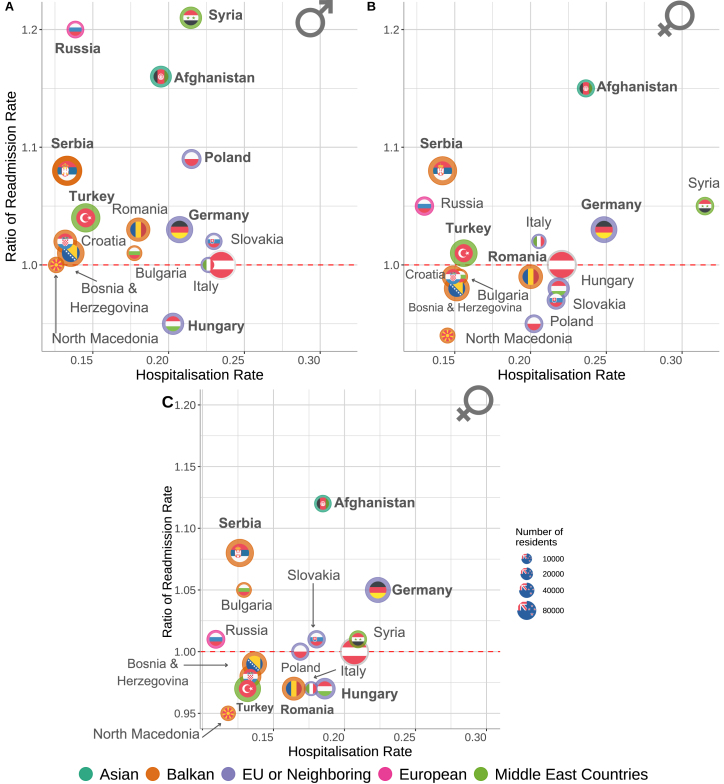
Hospitalization rate of cohorts with different countries of origin and ratio of readmission rate in the next year of each cohort and their (age, sex, region of residence, and month of hospital visit) matched cohort of Austrian patients (a) males, (b) females, (c) females — hospitalization rate without pregnancy-related hospital stays. Countries in bold exhibit statistically significantly different readmission rates compared to matched Austrian counterparts. The size of the flags is proportional to the number of residents in Austria of each country of origin. The circle’s colour around the flags depicts an area of the country (with respect to Austria). (For interpretation of the references to colour in this figure legend, the reader is referred to the web version of this article.)

A crucial element observed is that we found similar hospitalization and readmission rates for countries from the same area. For example, most Balkan countries, like Serbia, Croatia, Bosnia and Herzegovina, and North Macedonia, have comparable hospitalization rates, Fig. 6. Thus, hospitalization and readmission rates capture a collective behaviour observed for different groups of migrants in a hosting country.

Excluding pregnancy-related hospital stays for female patients, we found that the highest hospital rate was for German patients (0.22), while the lowest was still for patients from Russia (0.11), Fig. 6c. The hospitalization rate for Austrian female patients is 0.21. Countries like Bosnia and Herzegovina, Germany, Croatia, Serbia, Italy and Hungary had similar estimates for hospitalization rates (including and excluding pregnancy-related hospital stays), where the difference was less than 15%. The biggest difference between these two hospitalization rates we identified for females from Syria, where 38% of all hospital patients go to a hospital because of a pregnancy. However, in the case of Austria, only 9.5% of female patients had only pregnancy-related hospital stays.

Comparing baseline characteristics across the top ten countries, based on the number of patients, we found that almost in all cases, non-Austrian patients had a smaller number of diagnoses, fewer hospital stays and fewer hospital days in comparison with their matched Austrian peers, considering age, sex, the month of visit, region of residence (Table 2 and SI Figure 23).

We compared the readmission rate of non-Austrian patients with matched Austrian peers and found that the biggest differences between rates are for patients from Afghanistan, for females and males. Patients from Turkey, Germany, and Serbia have higher readmission rates for females and males. While patients from Bosnia & Herzegovina, Romania, Croatia, Poland, and Slovakia have different rates for males and females, males have readmission rates comparing their Austrian peers, and this effect is the opposite for female patients. Hungarian female and male patients showed lower readmission rates than matched Austrian patients.

**Table 2:** Baseline characteristics table for selected countries of origin (those with the largest patient numbers) and their matched Austrian cohorts (matched by age, gender, month of visit, and region of residence). We tested whether differences between non-Austrians and their matched Austrian cohorts – specifically in the number of diagnoses, hospital stays, and hospital days – were statistically significant ( ${}^{*}p<0.05$, ${}^{**}p<0.01$, ${}^{***}p<0.001$). The p-values refer to comparisons between each non-Austrian group and its respective matched Austrian cohort. The readmission probability (RP) (second to the last column) shows the probability of having another hospital visit in the following year, while the last column, Ratio, shows the ratio of this probability and the readmission probability of the matched cohort of Austrian patients. A comprehensive table including all countries of origin can be found in Supplementary Information, Table 3.

Country	ISO2	Sex	Number of Diagnoses		Number of Hospital Stays		Number of Hospital Days		Total Number of Hospital Stays	RP	Ratio of RP
				Ratio		Ratio		Ratio			
Turkey	TR	Male	3.00	$0.91^{***}$	6.51	0.96	30.8	$0.84^{***}$	33 025	0.45	1.04
		Female	2.80	$0.92^{***}$	5.22	$0.84^{***}$	22.8	$0.72^{***}$	42 898	0.40	1.01
Germany	DE	Male	3.04	$0.82^{***}$	6.39	$0.89^{***}$	33.3	$0.83^{***}$	33 186	0.49	1.03
		Female	2.70	$0.81^{***}$	5.37	$0.84^{***}$	25.6	$0.75^{***}$	39 154	0.43	1.03
Serbia	RS	Male	3.41	0.93	7.53	0.95	40.9	0.99	33 162	0.52	1.08
		Female	3.14	$0.93^{*}$	6.43	$0.89^{**}$	33.0	$0.9^{**}$	40 161	0.47	1.08
Bosnia & Herzegovina	BA	Male	3.05	$0.84^{***}$	6.52	$0.91^{***}$	32.9	$0.82^{***}$	24 322	0.47	1.01
		Female	2.66	$0.81^{***}$	5.41	$0.83^{***}$	24.7	$0.74^{***}$	26 632	0.41	0.98
Romania	RO	Male	2.23	$0.77^{***}$	4.52	$0.83^{***}$	22.9	$0.77^{***}$	11 600	0.38	1.03
		Female	2.31	$0.8^{***}$	3.65	$0.7^{***}$	16.7	$0.62^{***}$	23 676	0.35	0.99
Croatia	HR	Male	2.92	$0.81^{***}$	5.83	$0.81^{***}$	31.0	$0..81^{***}$	15 544	0.47	1.02
		Female	2.56	$0.79^{***}$	5.22	$0.81^{***}$	23.6	$0.71^{***}$	16 463	0.41	0.99
Hungary	HU	Male	2.11	$0.73^{***}$	4.89	$0.87^{**}$	17.8	$0.58^{***}$	8913	0.36	0.95
		Female	2.23	$0.78^{***}$	3.98	$0.76^{***}$	15.8	$0.6^{***}$	15 470	0.34	0.98
Poland	PL	Male	2.83	$0.87^{***}$	7.22	1.06	35.4	0.97	12 003	0.46	1.09
		Female	2.29	$0.76^{***}$	5.63	0.93	23.3	$0.78^{***}$	13 535	0.36	0.95
Afghanistan	AF	Male	2.09	$0.85^{***}$	3.17	$0.67^{***}$	15.6	$0.62^{***}$	9802	0.36	1.16
		Female	2.57	$0.94^{**}$	4.08	$0.87^{***}$	17.4	$0.72^{**}$	9410	0.37	1.15
Slovakia	SK	Male	2.11	$0.74^{***}$	4.07	$0.69^{***}$	21.2	$0.71^{***}$	4760	0.37	1.02
		Female	2.20	$0.78^{***}$	3.81	$0.7^{***}$	16.8	$0.62^{***}$	11 973	0.35	0.97

To determine whether our findings are influenced by certain characteristics of the population of origin (such as the ratio of female residents, age structure, population size, cohort size of patients, and the size of the diaspora), we analysed the correlation between these factors and hospitalization and readmission rates. We did not find any significant correlation that could bias our findings (SI Figures 31 and 32).

### 3.2 Prevalence of first and all diagnoses of Austrians and non-Austrians

The information on the specific hospital departments or units within a hospital where patients are initially admitted can tell us what kind of care was provided and the nature of patient needs (SI Figure 17). Internal Medicine is the most frequent hospital department where female and male patients are admitted for initial hospital stay (32.1% of all males and 28.7% of females). Paediatrics is the most frequent for both sexes, aged 0 to 19 years old. The initial hospital visit of female patients aged 20–39 is in the Obstetrics and Gynecology section. Section Surgery is the most frequent for males 20 to 49 years old and females 40 to 59 years old. Internal medicine is the most common hospital department for male patients aged more than 50 years old and female patients older than 60.

**Fig. 7:**
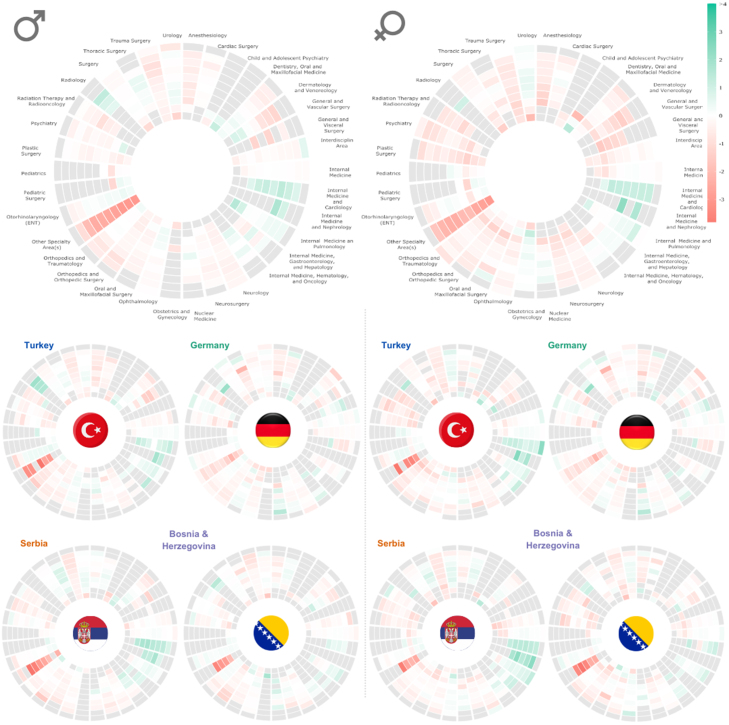
The top two graphs depict the ratio of prevalence of each hospital department of a first hospital visit in each 10-year age group (each circle) for non-Austrian nationality patients and Austrian nationality patients, males on the right side and females on the left (log scale). The bottom graphs show the results of four specific countries, with males on the right side and females on the left (log scale). The grey colour indicates a stratum without patients in that hospital department.

We found substantial differences in prevalences from some hospital departments by analysing the ratio of prevalences of patients with nationalities different from Austrian and the corresponding stratum of Austrian patients, Fig. 7. For instance, Other Specialty Areas and General and Vascular Surgery sections have lower prevalence in non-Austrian cohorts across all age groups. Meanwhile, internal medicine and cardiology, internal medicine and nephrology, internal medicine, haematology and oncology, and radiology are more often hospital departments of initial admission in non-Austrian patients. We found similar patterns when we analysed this on a country level for countries like Turkey, Serbia, Bosnia, and Herzegovina (Fig. 7). However, this is less prominent in Germany, and differences with the Austrian population are minor than in other non-Austrian populations.

**Fig. 8:**
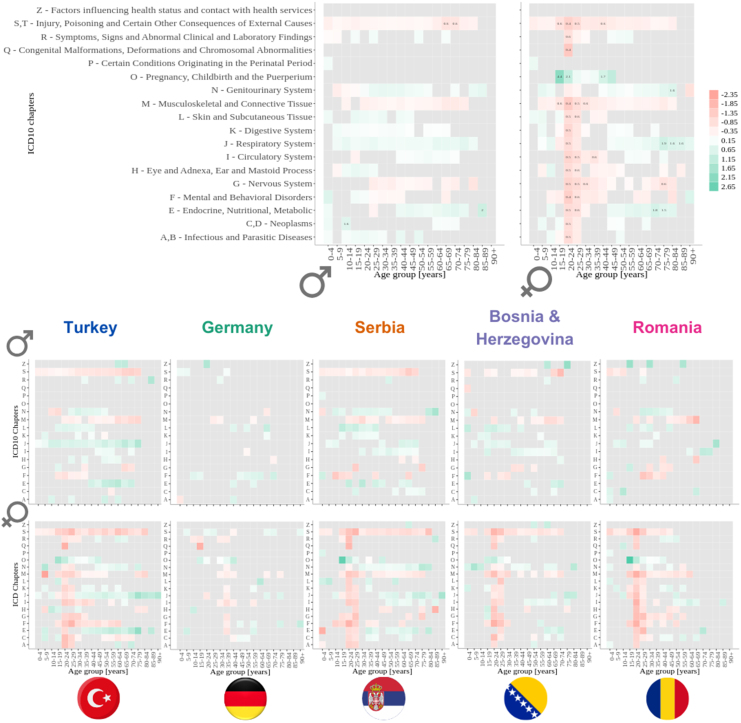
The top two graphs depict the ratio of prevalence of each type of diagnosis in each age group, non-Austrians/Austrians for the initial hospital stay, males on the right side, and females on the left (log scale). The bottom graphs show the results of four specific countries (the four largest groups of non-Austrian patients — Turkey, Germany, Serbia and Bosnia and Herzegovina), with males on the top and females on the bottom (log scale). The grey colour indicates a stratum without patients in that hospital department or that a certain probability of Non-Austrians is not significantly different from the corresponding probability of Austrians (p-value >=0.05).

Primary and secondary diagnoses were classified using level-4 ICD10 codes (the global standard for medical coding), ensuring consistency across healthcare systems (World Health Organization, 2019). The International Classification of Diseases (ICD) is a system produced by the World Health Organization that is crucial for tracking health trends and compiling international health statistics (details in the Methods section). To compare the most common reasons for initial hospital admissions, we calculated the prevalence of each ICD10 chapter of the primary diagnoses for each age group and sex for the Austrian and non-Austrian populations (SI Table 2 and Fig. 8). The primary diagnosis encodes the main reason for hospital admission. To compare these probabilities for different cohorts, we used the two-proportions z-test. The null hypothesis is that the corresponding probabilities for non-Austrian and Austrian patients are the same. We plotted the prevalence ratio of different ICD Chapters for non-Austrian and Austrian patients. However, we only plotted the ratio for cases where prevalences were significantly different, namely p_value<0.05 (the same results for all ICD10 codes except pregnancy-related diagnoses are shown in SI, Figure 14).

The ratio between the prevalence of hospital diagnoses chapters between Austrian and non-Austrians ranges from 0.36 to 6.67. The most common ICD Chapter where non-Austrians have significantly smaller probability compared to Austrians are injury, poisoning and certain other consequences of external causes (S,T) and musculoskeletal and connective tissue (M). The respiratory system (J) and genitourinary system (N) were among the most common ICD Chapters, for which non-Austrians have a significantly higher probability than Austrians. Non-Austrian female patients in age groups 20 to 39 years old have a significantly smaller probability for all ICD Chapters, except for pregnancy, childbirth and the puerperium (ICD Chapter O). We found the same pattern for countries like Turkey, Serbia, Bosnia & Herzegovina, and Romania. While this is not the case for female patients with German nationality, Fig. 8.

**Fig. 9:**
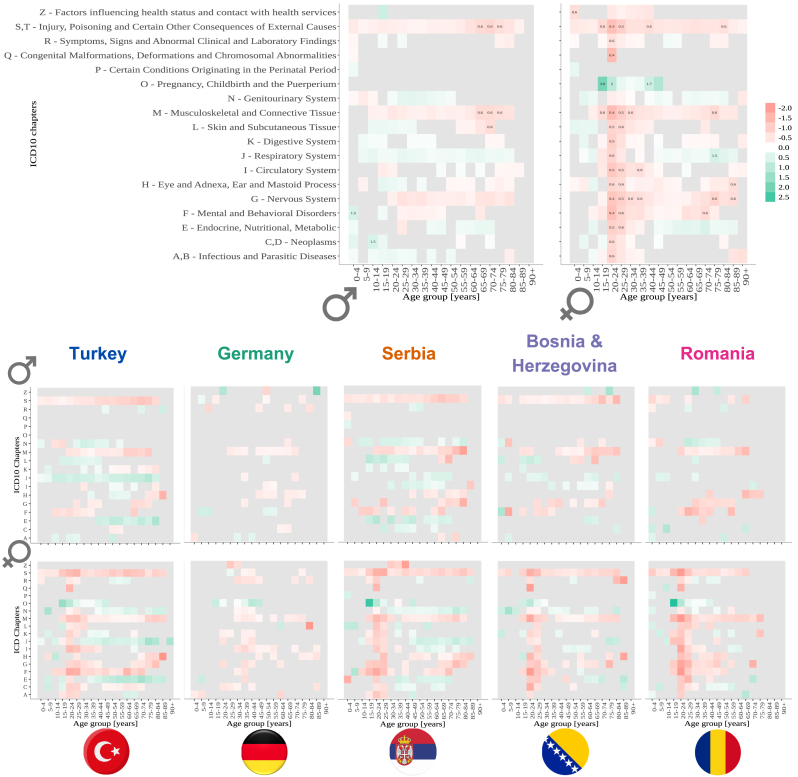
The top two graphs depict the ratio of prevalence of each type of diagnosis in each age group, non-Austrians/Austrians for all hospital stays, males on the right side, and females on the left (log scale). The bottom graphs show the results of four specific countries(the four largest groups of non-Austrian patients — Turkey, Germany, Serbia and Bosnia and Herzegovina), with males on the top and females on the bottom (log scale). The grey colour indicates a stratum without patients in that hospital department or that a certain probability of Non-Austrians is not significantly different from the corresponding probability of Austrians (p-value >=0.05).

Similar patterns to the diagnoses of the initial hospital admissions were found in the ratio between the prevalence of hospital diagnoses chapters between Austrian and non-Austrians for all hospital admissions Fig. 9. Concerning all hospital diagnoses, non-Austrian patients were more likely to be diagnosed with diseases of the respiratory (ICD chapter J) and genitourinary (N) system, next to endocrine, nutritional and metabolic diseases (E). However, diseases of the musculoskeletal system and connective tissue (M), injuries and poisonings (S, T) and mental and behavioural disorders (F) were used less often as primary hospital diagnoses in non-Austrians than in Austrians.

## 4 Discussion

In this extensive medical claims data analysis, we investigated differences in the usage of the hospital system by Austrians compared to non-Austrians. We focused on hospitalization rates and types of diagnoses to gauge how Austrians use the Austrian healthcare system compared to non-Austrians. Analysing a comprehensive medical claims data set with information on patients’ nationalities, we found that almost all nationalities, except for Germans, have lower hospitalization rates than Austrians. An exception is the hospitalization rate for non-Austrian women, which is higher than their Austrian counterparts. However, these hospitalizations are mostly comprised of admissions related to childbirth. We also saw clear differences in the diseases/diagnoses migrants and/or Austrians are admitted to the hospital.

Three critical results for medical care were derived from the analysed data. Almost all nationalities have lower hospitalization rates than Austrians (except for Germans living in Austria). In Austria, almost 20% of the population has a non-Austrian nationality but only comprises 9.4% of the hospital patients and 9.79% hospital nights (Prieto-Curiel et al., 2024). Although Germany is in second place regarding total patient count, it does lead in terms of the highest number of hospital stays. Male patients of all nationalities have lower hospitalization rates. On average, migrants go to the hospital less often (and potentially later in the development of a medical condition) as the readmission rate is higher in migrants than in Austrians (Milne and Clarke, 1990, Bensken et al., 2021). At the same time, female patients from Germany, Afghanistan, and Syria have higher hospitalization rates in comparison to Austrians. However, in the case of hospitalization rates without pregnancy-related hospital stays, only German female patients have a higher hospitalization rate. Non-Austrian women between the ages of 20 and 39 years were less likely to be admitted for any diagnosis other than those related to obstetrics (childbirth). This might indicate that non-Austrian women are either healthier than Austrian women, or it might indicate a hesitance to use the medical system, leading to worse medical care for non-Austrian women.

Our results show that almost all nationalities (except female German patients) have lower hospitalization rates. However, many nationalities also showed higher readmission rates, which may indicate that the “healthy migrant” effect does not explain the lower hospitalization rate. Instead, a lower hospitalization rate may be present because of barriers to accessing the healthcare system and may signal differences in timely access to preventive care. Hence, when migrants enter the healthcare system, there is a higher probability they will come again (higher readmission rate). This could also indicate that their initial hospital visits occur at more advanced stages of illness, resulting in more severe cases and missed opportunities for timely prevention and intervention. Furthermore, the lack of data on the quality of care and treatments provided raises the possibility that migrants may face discrimination within healthcare services. Males from Syria, Russia, Afghanistan, Serbia, Poland, Turkey, and Germany have significantly higher readmission rates compared to matched Austrians. In females, the readmission rate differs greatly if we include or exclude pregnancy-related hospital stays. Namely, female patients with Turkish nationality have significantly higher readmission rates compared to Austrians if we include pregnancy. However, in the case of hospitalization without pregnancy-related stays, they have significantly lower readmission rates. At the same time, having a lower hospitalization rate and significantly lower readmission rate in female patients of Turkish nationality may indeed be driven by the “healthy migrant” effect. Female patients from Romania (and Hungary in case of stays without pregnancy-related hospitalizations) showed significantly lower readmission rates compared to Austrian females. In this case, the finding may be explained by the fact that these two countries are neighbouring countries, meaning these patients might also seek healthcare in their own country as EU citizens can access healthcare in other EU countries using their resident country’s healthcare insurance (European Commission, 2004).

Germans exhibit the most similar pattern in terms of admitted hospital department, first diagnosis, and all hospital diagnoses compared to Austrians. Except for Germans, we see clear differences in the hospital departments and diseases/diagnoses for which non-Austrians and Austrians are being admitted to the hospital. Non-Austrians of both sexes are more likely to be administrated in the Department of Radiology as well as all Departments of Internal Medicine. Female patients between the ages of 20 and 39 years frequent the Department of Obstetrics and Gynecology more often than their Austrian, age-matched counterparts. Moreover, there are some differences concerning the diagnoses for which the patients are admitted to the hospital. Young female non-Austrians are more often diagnosed with conditions related to Gynecology and Obstetrics, which is in line with birth rates being higher amongst non-Austrians (Statistik Austria, 2022).

In non-Austrians of both sexes, the first diagnosis of a specific person was more likely to be from the chapters diseases of the genitourinary system (ICD chapter N), skin and subcutaneous tissue (L) and internal medicine (e.g. Diseases of the digestive System (K), diseases of the respiratory system (J), diseases of the circulatory system (I) and endocrine, nutritional and metabolic diseases (E)). In terms of all coded hospital diagnoses, non-Austrian patients were more likely to be diagnosed with diseases of the respiratory system (J), diseases of the genitourinary system (N) and endocrine, nutritional and metabolic diseases (E). For instance, Turkish people of both sexes were more often admitted to the hospital due to respiratory issues. A slightly higher prevalence of smoking in Turkey (27.3%) than in Austria (22.4%) might partly explain the discrepancy (Eurostat, 2022).

Diseases of the musculoskeletal system and connective tissue (M), injury, poisoning and certain other consequences of external causes (S,T) and mental and behavioural disorders (F) were much less coded in non-Austrians than Austrians. Language and cultural barriers might hinder non-Austrians from seeking help for mental and behavioural disorders. It has been shown that difficulties in understanding and articulating emotional stress hinder help-seeking for mental conditions and that there is a clear association between a lack of language proficiency and underuse of psychiatric help (Ohtani et al., 2015). This is especially troubling for refugees, who are more likely to suffer from psychiatric diagnoses such as post-traumatic stress disorder, depression, anxiety, and panic disorder (Bhui et al., 2003, Mollica et al., 2001, Sønderskov et al., 2021, Caroppo et al., 2023).

This study’s main strength lies in its extensive unique data. It comprehensively captures in-hospital data for approximately four million individuals. However, there are certain limitations to consider, primarily stemming from data quality and accessibility constraints. The data does not include information on outpatient medical services, prescriptions, or lifestyle factors. Furthermore, the absence of socioeconomic data for the individual patients means that the potential effects of socioeconomic factors remain unknown. Importantly, the dataset only captures individuals who accessed hospital care and received a diagnosis, which limits the ability to draw conclusions about those who may have unmet healthcare needs but did not engage with the healthcare system. Yet, results regarding hospital departments may be biased due to the different demographics of mostly Viennese (bigger) hospitals. Additionally, one more limitation of streaming data is that the database covers only five years and that we did not include patients with more than one nationality. We must emphasize that another potential limitation of this study lies in the estimates of “years of life” (YoL). We do not have data on the number of days Austrians spend in Austria, so we assumed that YoL for all Austrians is 1. We do not have data on days spent for other nationalities for 2019, but only for 2023. This means that our estimates of YoL for 2019 are based on data from 2023. Additionally, the flow estimates of the non-Austrian resident population assume a stable flow between 2023 and 2019, which is not valid in some cases, such as in Syria.

Non-Austrians use the healthcare system less but are more likely to be readmitted, potentially due to more severe illness. Non-Austrians of both sexes are less likely to be diagnosed with an ICD10 diagnosis from chapter mental and behavioural disorders (F) and more often hospitalized with diseases from the ICD10 chapters of internal medicine, genitourinary diseases, and skin disorders. A prominent healthcare gap concerns young non-Austrian women, who are much less likely to go to a hospital than their Austrian peers. Similarly, results of the Refugee Health and Integration Survey (ReHIS) in Austria, published in 2019, indicated the refugee’s self-rated health to be below the self-rated health of Austrians. Listed barriers were long waiting lists, lack of knowledge about doctors, and a language barrier (Kohlenberger et al., 2019). Less access to healthcare for non-Austrians is critical in terms of less preventative healthcare such as vaccinations, higher rates of communicable diseases or chronic diseases aggravating due to the lack of treatment. This might not only impact a person’s physical health but also mental health and quality of life.

Early prevention plays a crucial role in maintaining an individual’s well-being and public health. This is especially crucial considering that non-Austrians in our cohort are, on average, 13.9 years younger than Austrians. Prevention involves taking proactive measures to identify and mitigate potential health risks before they develop into more severe conditions. This approach is particularly significant in managing chronic diseases, where early intervention can dramatically reduce the severity and progression of the illness. Targeted research on chronic diseases can provide strategic, data-based solutions for preventing diseases, addressing some of the critical issues associated with this demographic evolution (World Health Organization, 2015). Furthermore, the findings’ policy implications regarding migrant populations in Austria may provide valuable insights for policymakers.

Taken together, the different patterns we found in hospitalization and readmission rates for people from countries other than Austria show that most of the migrant population using fewer healthcare systems cannot be driven only by controversial hypotheses of “healthy migrant” or only due to cultural barriers. Instead, utilization of the healthcare system in migrant populations has to be studied separately for different countries, as we appear to see different healthcare utilization patterns in diverse cohorts of migrants.

Future work should be dedicated to further investigating healthcare utilization and the comorbidities and multimorbidity patterns of patients of different nationalities. Future projects should develop strategies to reduce the barrier for non-Austrians to use the healthcare system, especially the outpatient system. Examples could be better translation services in hospitals and doctor’s offices using web-based interpretation services, courses on how to navigate the Austrian healthcare system, particularly specialist care, or language courses to bridge the language gap.

## CRediT authorship contribution statement

**Elma Dervić:** Writing – review & editing, Writing – original draft, Visualization, Software, Methodology, Formal analysis, Data curation, Conceptualization. **Ola Ali:** Writing – review & editing, Formal analysis, Data curation. **Carola Deischinger:** Writing – review & editing, Writing – original draft. **Rafael Prieto-Curiel:** Writing – review & editing, Supervision, Methodology, Conceptualization. **Rainer Stütz:** Writing – review & editing. **Ellenor Mittendorfer-Rutz:** Writing – review & editing. **Peter Klimek:** Writing – review & editing, Supervision, Methodology, Conceptualization.

## Ethics

We made secondary use of a research database containing medical claims records, which is securely managed by the Federal Ministry of Health. It is important to note that measures have been implemented to guarantee the anonymity of individuals within this database. It is a consolidated research database accessible only to authorized partners who adhere to stringent data protection policies. Our use of this data is conducted in collaboration with the data provider and follows established agreements.

The data in this database do not include any personal identifiers, such as names, postal codes, or dates of birth. Additionally, all members of our research team have committed to maintaining confidentiality and complying with relevant data protection regulations through a signed agreement.

## Declaration of competing interest

The authors declare that they have no known competing financial interests or personal relationships that could have appeared to influence the work reported in this paper.

## Data Availability

The raw and processed patient data are not available due to privacy laws. The dataset is safeguarded by the Austrian Federal Ministry of Health and made accessible to research institutions under strict data protection regulations. To gain access to this data, researchers have to find individual arrangements with the Austrian Federal Ministry of Health.

## Appendix A Supplementary data

MMC S1 SI: Healthcare Utilization Patterns Among Migrant Populations: Increased Readmissions Suggest Poorer Access.

## References

[b1] Assi R., Özger-İlhan S., İlhan M.N. (2019). Health needs and access to health care: the case of Syrian refugees in Turkey. Public Health.

[b2] Backman Mikaela, Lopez Esteban, Rowe Francisco (2018). Career trajectories and outcomes of forced migrants in Sweden: Self-employment, employment or persistent inactivity?. Small Bus. Econ..

[b3] Bensken Wyatt P., Alberti Philip M., Koroukian Siran M. (2021). Health-related social needs and increased readmission rates: findings from the nationwide readmissions database. J. Gen. Intern. Med..

[b4] Bhui Kamaldeep, Abdi Abdisalama, Abdi Mahad, Pereira Stephen, Dualeh Mohammed, Robertson David, Sathyamoorthy Ganesh, Ismail Hellena (2003). Traumatic events, migration characteristics and psychiatric symptoms among somali refugees: Preliminary communication. Soc. Psychiatry Psychiatr. Epidemiol..

[b5] Blinderman Craig D., Homel Peter, Billings J. Andrew, Tennstedt Sharon, Portenoy Russell K. (2009). Symptom distress and quality of life in patients with advanced chronic obstructive pulmonary disease. J. Pain Symptom Manage..

[b6] Brandenberger Julia, Tylleskär Thorkild, Sontag Katrin, Peterhans Bernadette, Ritz Nicole (2019). A systematic literature review of reported challenges in health care delivery to migrants and refugees in high-income countries-the 3C model. BMC Public Health.

[b7] Buchcik Johanna, Borutta Jana, Nickel Stefan, von dem Knesebeck Olaf, Westenhöfer Joachim (2021). Health-related quality of life among migrants and natives in Hamburg, Germany: An observational study. J. Migr. Heal..

[b8] Caroppo Emanuele, Calabrese Carmela, Mazza Marianna, Rinaldi Alessandro, Coluzzi Daniele, Napoli Pierangela, Sapienza Martina, UOC Salute Mentale working group Monfrinotti Italo, Porfiri Maurizio, De Lellis Pietro (2023). Migrants’ mental health recovery in Italian reception facilities. Commun. Med..

[b9] Chiarenza Antonio, Dauvrin Marie, Chiesa Valentina, Baatout Sonia, Verrept Hans (2019). Supporting access to healthcare for refugees and migrants in European countries under particular migratory pressure. BMC Health Serv. Res..

[b10] Constant Amelie, Massey Douglas S. (2003). Self-selection, earnings, and out-migration: A longitudinal study of immigrants to Germany. J. Popul. Econ..

[b11] Dervic Elma, Deischinger Carola, Haug Nils, Leutner Michael, Kautzky-Willer Alexandra, Klimek Peter (2021). The effect of cardiovascular comorbidities on women compared to men: Longitudinal retrospective analysis. JMIR Cardio.

[b12] Dunlavy Andrea, Cederström Agneta, Katikireddi Srinivasa Vittal, Rostila Mikael, Juárez Sol P. (2022). Investigating the salmon bias effect among international immigrants in Sweden: a register-based open cohort study. Eur. J. Pub. Health.

[b13] Dupre Matthew E., Gu Danan, Vaupel James W. (2012). Survival differences among native-born and foreign-born older adults in the United States. PLoS One.

[b14] European Commission (2004). https://ec.europa.eu/commission/presscorner/detail/en/IP_04_390.

[b15] Eurostat (2019). https://ec.europa.eu/eurostat/documents/3217494/10166544/KS-02-19%E2%80%91681-EN-N.pdf/c701972f-6b4e-b432-57d2-91898ca94893.

[b16] Eurostat,, 0000. Smoking of tobacco products by sex, age and country of citizenship, https://ec.europa.eu/eurostat/databrowser/view/hlth_ehis_sk1c/default/table?lang=en.

[b17] Gil-Salmerón Alejandro, Katsas Konstantinos, Riza Elena, Karnaki Pania, Linos Athena (2021). Access to healthcare for migrant patients in Europe: healthcare discrimination and translation services. Int. J. Environ. Res. Public Heal..

[b18] Gnisci Donata (2008).

[b19] Haug Nina, Deischinger Carola, Gyimesi Michael, Kautzky-Willer Alexandra, Thurner Stefan, Klimek Peter (2020). High-risk multimorbidity patterns on the road to cardiovascular mortality. BMC Med..

[b20] Holz Manuel (2022). Health inequalities in Germany: Differences in the ‘Healthy migrant effect’of European, non-European and internal migrants. J. Ethn. Migr. Stud..

[b21] Ichou Mathieu, Wallace Matthew (2019). The healthy immigrant effect. Demogr. Res..

[b22] Kennedy Steven, Kidd Michael P., McDonald James Ted, Biddle Nicholas (2015). The healthy immigrant effect: patterns and evidence from four countries. J. Int. Migr. Integr..

[b23] Kohlenberger Judith, Buber-Ennser Isabella, Rengs Bernhard, Leitner Sebastian, Landesmann Michael (2019). Barriers to health care access and service utilization of refugees in Austria: Evidence from a cross-sectional survey. Heal. Policy.

[b24] Kristensen Søren Rud, Bech Mickael, Quentin Wilm (2015). Is the readmission rate a valid quality indicator? A review of the evidence. PLoS One.

[b25] Kullamaa Lembe, Reile Rainer (2023). Socio-demographic and regional differences in unmet healthcare needs among migrants in Europe. Plos One.

[b26] Lebano Adele, Hamed Sarah, Bradby Hannah, Gil-Salmerón Alejandro, Durá-Ferrandis Estrella, Garcés-Ferrer Jorge, Azzedine Fabienne, Riza Elena, Karnaki Pania, Zota Dina (2020). Migrants’ and refugees’ health status and healthcare in Europe: a scoping literature review. BMC Public Health.

[b27] Lewis Eldrin F., Li Yanhong, Pfeffer Marc A., Solomon Scott D., Weinfurt Kevin P., Velazquez Eric J., Califf Robert M., Rouleau Jean-Lucien, Kober Lars, White Harvey D. (2014). Impact of cardiovascular events on change in quality of life and utilities in patients after myocardial infarction: a VALIANT study (valsartan in acute myocardial infarction). JACC: Hear. Fail..

[b28] Ma Chao, Qu Zhaopeng, Xu Zimeng (2020). Internal migration and mental health: An examination of the healthy migration phenomenon in China. Popul. Res. Policy Rev..

[b29] Milne Ruairidh, Clarke Aileen (1990). Can readmission rates be used as an outcome indicator?. BMJ: Br. Med. J..

[b30] Mollica Richard F., Sarajlić Narcisa, Chernoff Miriam, Lavelle James, Vuković Iris Sarajlić, Massagli Michael P. (2001). Longitudinal study of psychiatric symptoms, disability, mortality, and emigration among bosnian refugees. Jama.

[b31] Ng Edward (2011). The healthy immigrant effect and mortality rates. Health Rep..

[b32] Niva Venla, Horton Alexander, Virkki Vili, Heino Matias, Kosonen Maria, Kallio Marko, Kinnunen Pekka, Abel Guy J, Muttarak Raya, Taka Maija (2023). World’s human migration patterns in 2000–2019 unveiled by high-resolution data. Nat. Hum. Behav..

[b33] Ohtani Ai, Suzuki Takefumi, Takeuchi Hiroyoshi, Uchida Hiroyuki (2015). Language barriers and access to psychiatric care: a systematic review. Psychiatr. Serv..

[b34] Ortensi Livia Elisa, Ambrosetti Elena (2022). Even worse than the undocumented? Assessing the refugees’ integration in the labour market of Lombardy (Italy) in 2001–2014. Int. Migr..

[b35] Pattillo M., Stieglitz S., Angoumis K., Gottlieb N. (2023). The experience of racism against migrants in healthcare in europe: a scoping review. Eur. J. Public Heal..

[b36] Prieto-Curiel Rafael, Ali Ola, Dervic Elma, Karimi Fariba, Omodei Elisa, Stütz Rainer, Heiler Georg, Holovatch Yurij (2024). The diaspora model for human migration. PNAS Nexus.

[b37] Prieto-Curiel Rafael, Quiñones Domínguez Mauricio, Lora Eduardo, O’Clery Neave (2022). Mobility between Colombian cities is predominantly repeat and return migration. Comput. Environ. Urban Syst..

[b38] Pugh Jacqueline, Penney Lauren S., Noël Polly H., Neller Sean, Mader Michael, Finley Erin P., Lanham Holly J., Leykum Luci (2021). Evidence based processes to prevent readmissions: more is better, a ten-site observational study. BMC Health Serv. Res..

[b39] Quinn Sandra Crouse, Kumar Supriya (2014). Health inequalities and infectious disease epidemics: a challenge for global health security. Biosecurity Bioterrorism: Biodefense Strat. Pr. Sci..

[b40] Razum Oliver, Zeeb Hajo, Akgün H. Seval, Yilmaz Selma (1998). Low overall mortality of Turkish residents in Germany persists and extends into a second generation: merely a healthy migrant effect?. Trop. Med. Int. Health.

[b41] Rousseau Cécile, Frounfelker Rochelle L. (2019). Mental health needs and services for migrants: an overview for primary care providers. J. Travel. Med..

[b42] Sîrbu Alina, Andrienko Gennady, Andrienko Natalia, Boldrini Chiara, Conti Marco, Giannotti Fosca, Guidotti Riccardo, Bertoli Simone, Kim Jisu, Muntean Cristina Ioana (2020). Human migration: the big data perspective. Int. J. Data Sci. Anal..

[b43] Sønderskov Kim M., Dinesen Peter T., Hansen Bertel T., Østergaard Søren D., Danckert Bolette (2021). Terrorism in the country of origin is linked to deterioration in the mental health of refugees. Nat. Hum. Behav..

[b44] Statistik Austria (2022). https://www.statistik.at/fileadmin/publications/Migration_Integration_2022.pdf.

[b45] Statistik Austria (2024). https://www.statistik.at/en/statistics/population-and-society/population/migration-and-naturalisation/naturalisation.

[b46] Statistik Austria (2024). https://www.statistik.at/datenbanken/statcube-statistische-datenbank.

[b47] United Nations Department of Economic and Social Affairs (DESA) (2020).

[b48] Vintila Daniela, Lafleur Jean-Michel (2020). Migration and Social Protection in Europe and Beyond (Volume 1) Comparing Access to Welfare Entitlements.

[b49] Wallace Matthew, Kulu Hill (2015). Mortality among immigrants in England and Wales by major causes of death, 1971–2012: a longitudinal analysis of register-based data. Soc. Sci. Med..

[b50] Ward Markia, Kristiansen Maria, Sørensen Kristine (2019). Migrant health literacy in the European union: A systematic literature review. Heal. Educ. J..

[b51] World Health Organization (2015).

[b52] World Health Organization (2019). https://www.who.int/standards/classifications/classification-of-diseases.

[b53] World Health Organization (2022).

